# DynaMut: predicting the impact of mutations on protein conformation, flexibility and stability

**DOI:** 10.1093/nar/gky300

**Published:** 2018-04-30

**Authors:** Carlos HM Rodrigues, Douglas EV Pires, David B Ascher

**Affiliations:** 1 Department of Biochemistry and Molecular Biology, Bio21 Molecular Science and Biotechnology Institute, University of Melbourne, Australia; 2 Instituto René Rachou, Fundação Oswaldo Cruz, Brazil; 3 Department of Biochemistry, University of Cambridge, UK

## Abstract

Proteins are highly dynamic molecules, whose function is intrinsically linked to their molecular motions. Despite the pivotal role of protein dynamics, their computational simulation cost has led to most structure-based approaches for assessing the impact of mutations on protein structure and function relying upon static structures. Here we present DynaMut, a web server implementing two distinct, well established normal mode approaches, which can be used to analyze and visualize protein dynamics by sampling conformations and assess the impact of mutations on protein dynamics and stability resulting from vibrational entropy changes. DynaMut integrates our graph-based signatures along with normal mode dynamics to generate a consensus prediction of the impact of a mutation on protein stability. We demonstrate our approach outperforms alternative approaches to predict the effects of mutations on protein stability and flexibility (*P*-value < 0.001), achieving a correlation of up to 0.70 on blind tests. DynaMut also provides a comprehensive suite for protein motion and flexibility analysis and visualization via a freely available, user friendly web server at http://biosig.unimelb.edu.au/dynamut/.

## INTRODUCTION

Proteins are dynamic macromolecules, whose function is intricately linked to their biological motions (1,2). We have shown previously that drug resistant and genetic disease mutations can both act through changes in protein conformational equilibria and dynamics (3–7). In order to fully understand the molecular consequences of a mutation it is, therefore, important to consider changes in protein dynamics. Despite their pivotal role, the computational cost of dynamics simulation has led to most structure-based approaches for assessing mutations effects on protein structure and function relying upon static structures.

Normal Mode Analysis (NMA) is a computational approach that approximates the dynamics of a system around a conformation through harmonic motion. This has been used to generate possible movements and therefore provide valuable insights into protein motions, and their accessible conformational repertoires. Previous studies have shown that NMA can be a powerful tool to analyze protein structure–function relationship (8) and to predict the effects of single-point mutations on protein stability (9). Many NMA methods have been proposed (10–14) to address the lack of easy to use interfaces that limited their use to those with specialist knowledge. However, these are limited to the analysis of protein structures and do not provide approaches to evaluate the effect of mutations within their pipelines.

To fill this gap, we introduce DynaMut, a web server that introduces the dynamics component to mutation analysis. This is achieved by implementing and integrating well established normal mode approaches with our graph-based signatures in a consensus predictor for protein stability changes upon mutation, which we show optimizes overall prediction performance.

DynaMut implements NMA through two different approaches, Bio3D (8) and ENCoM (9), providing rapid and simplified access to powerful and insightful analysis of protein motions. In addition, DynaMut also enables rapid analysis of the impact of mutations on a protein's dynamics and stability resulting from vibrational entropy changes. Integration of these two different approaches with other well-established methods and characteristics of the wild-type residue environment into a consensus prediction enables DynaMut to provide an accurate assessment of the impact of a mutation on protein stability, and provide a comprehensive suite for protein motion and flexibility analysis and visualization via an easy-to-use web interface (http://biosig.unimelb.edu.au/dynamut/).

## MATERIALS AND METHODS

### Data sets

In this work, we used the previously established S2648 dataset (15–18), derived from the ProTherm database (19). This dataset is comprised of 2648 different point-mutations across 131 globular proteins with experimentally determined structures whose impact on protein stability has been experimentally measured (602 stabilizing and 2046 destabilizing). The DynaMut training set comprises 2297 mutations randomly selected from the original dataset. A blind test set composed of 351 non-redundant mutations derived from the S2648 set was also compiled. This blind test set has been widely used in the literature (15–18), enabling direct comparative performance of methods that quantify the impact of mutations on the folding free energy.

Previous studies have reported performance comparisons of difference methods on predicting changes in folding free energy (ΔΔ*G*) using these datasets (20–22). Given the unbalanced nature of the original dataset, here we have considered the hypothetical reverse mutations (22) in order to build a more robust, balanced and self-consistent predictive method. The change in folding free energy is a thermodynamic state function, and it has been proposed that the change in folding free energy of a mutation from a wild-type protein to its mutant (ΔΔ*G*_WT→MT_) should be equivalent to the negative change in folding free energy of the hypothetical reverse mutation—from the mutant to the wild-type protein (–ΔΔ*G*_MT→WT_) (16,22–24). Including the hypothetical reverse mutations, our predictive model was trained using 4594 mutations and our blind test was comprised of 702 single-point mutations.

### Normal mode analysis

NMA allows the study of harmonic motions in a system, providing insights into its dynamics and accessible conformations. It has been widely used for studies of protein dynamics as an alternative to more computationally intensive molecular dynamics approaches (25–28). While molecular dynamics approaches provide motion trajectories for a given molecule over time, conformational fluctuations can be evaluated by NMA via superposition of normal modes (Eigenvectors) and their associated frequencies (Eigenvalues) (29). NMA can also use simplified representations of the protein structure, such as modeling the amino acids using their Cα atoms, reducing computational cost. NMA has been successfully applied to the study of the effects of mutations on protein dynamics, with ENCoM (9) including the nature of the amino acids in the protein as an extra layer of information to compute the effects of single-point mutations on the vibrational entropy (ΔS) and protein stability.

### Other structure-based approaches

Structure-based approaches to predict the impact of mutations on stability utilize protein structural information from the 3D space of a natively folded protein. Even though these structure-based methods are essentially based on the same structural data, they are built using broadly different, sophisticated, approaches, such as statistical potential function energy calculations, used in SDM (16) and structural pattern mining approaches such as mCSM-Stability (18). The consensus method DUET highlighted that these approaches were complimentary, and that their integration provided more accurate and reliable predictions (17). This has been used to provide invaluable insights into disease and drug resistance mutations, and help guide protein engineering efforts (30–39).

### DynaMut—consensus predictions

**Figure 1.**
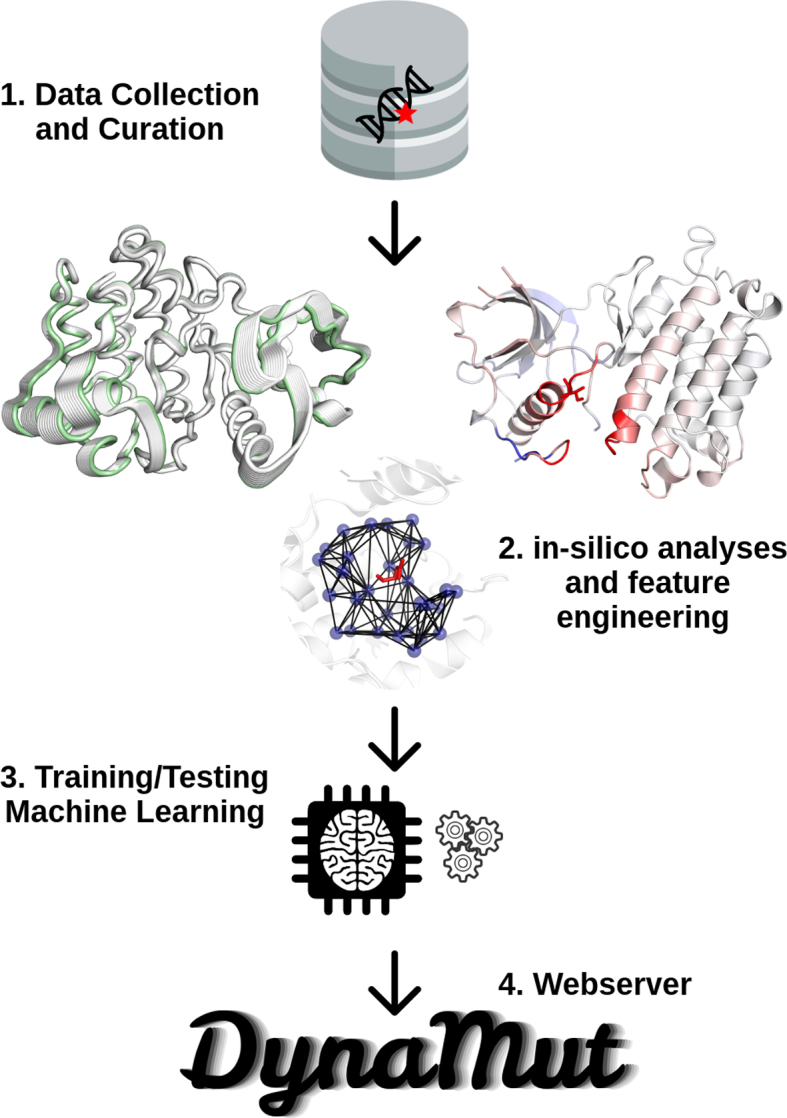
Methodology workflow. The DynaMut methodology can be divided into four steps. In step 1, data was collected from the previously established S2648 subset of mutations with experimental evidence from ProTherm. In step 2, DynaMut combines the effects of mutations on protein stability and dynamics calculated by Bio3D, ENCoM and DUET. In addition, DynaMut also includes a set of complementary information regarding the environment characteristics of the wild-type residue (e.g. relative solvent accessibility, residue depth and secondary structure) and the graph-based signatures generated by mCSM. All these features are used as evidence for training supervised learning algorithms in step 3. After evaluating the performance of the predictive model, the consensus prediction was integrated into the DynaMut web server.

Within DynaMut we have implemented a consensus estimate of changes upon mutation on protein folding free energy, which combines the effects of mutations on protein stability and dynamics calculated by Bio3D, ENCoM and DUET to generate an optimized and more robust predictor. Moreover, DynaMut includes a set of complementary information regarding the environment characteristics of the wild-type residue (*e.g*., relative solvent accessibility, residue depth and secondary structure) and graph-based signatures representing the wild-type structure. The graph-based signatures concept, used in the development of mCSM-Stability and to generate the consensus DUET predictions, has been widely applied to the study of protein structure, including protein–ligand interactions (40), and how mutations alter protein interactions with other molecules (23,24,41–43). These were supplied as evidence for training the consensus predictor using Random Forest (44). Figure 1 shows the workflow used to train the consensus predictions. The DynaMut consensus prediction was trained under 10-fold cross validation, and validated using the non-redundant blind test set (Supplementary Materials). The machine learning algorithm, evaluation procedures, performance metrics and details on the methods used on the consensus prediction are described in Supplementary Materials.

## WEB SERVER

We have implemented DynaMut as a user-friendly, freely available web server (http://biosig.unimelb.edu.au/dynamut/). The server front end was built using Bootstrap framework version 3.3.7, while the back-end was built in Python via the Flask framework (Version 0.12.2). It is hosted on a Linux server running Apache.

### Input

DynaMut can be used in two different ways, to either (1) analyze protein dynamics or (2) to analyze the effect of point mutations on protein dynamics and stability. For protein dynamics analysis (Supplementary Figure S1), the server requires the user to input a protein structure by either uploading a file in PDB format or by providing the four-letter accession code for any entry on the PDB database. In addition, users have the option to choose a specific force field, which is used to describe the molecular interactions within the structure for normal mode analysis. The force field options available are summarized in Supplementary Table S1 of Supplementary Materials.

Alternatively, for assessing the effects of mutations on protein dynamics and stability, two different input options are available (Supplementary Figure S2). The ‘Single mutation’ option requires the user to provide a PDB file or PDB accession code, the point mutation specified as a string containing the wild-type residue one-letter code, its corresponding residue number and the mutant residue one-letter code. The ‘Mutation list’ option allows users to upload a list of mutations in a file for batch processing. For both input options the user is also asked to specify the chain identifier in which the wild-type residue is located.

In order to assist users to submit their jobs for analysis and predictions, sample submission entries are available in both submission pages and a help page is available via the top navigation bar.

### Output

For the analysis of protein dynamics, the results are displayed in four tabs. In the first tab (Supplementary Figure S3), porcupine plots show the trajectory of movement according to the first non-trivial mode of the molecule. The second tab (Supplementary Figure S4) allows users to visualize the non-trivial modes generated, including an animated plot that describes the motion of the molecule. Visual representations of deformation energy and atomic fluctuation are displayed on the third tab (Supplementary Figure S5). Finally, the last tab shows the cross-correlation between residue movements as both a correlation matrix and the 3D structure of the submitted protein (Supplementary Figure S6).

The mutational analysis results are also split into tabs to enable users to easily navigate the different analyses available for evaluating the effects of mutations on protein stability and dynamics. For the ‘Single mutation’ option, the server outputs the predicted change in stability (in kcal/mol), along with the variation in entropy energy between wild-type and mutant structures (in kcal/mol/K) in the first tab (Supplementary Figure S7). For comparison purposes, in a separate panel the changes in stability calculated by structure-based methods are shown (16–18). DynaMut enables visualization of the non-covalent molecular interactions calculated by Arpeggio (45) (Supplementary Table S2, Supplementary Figure S8) and deformation energies and atomic fluctuations of wild-type and mutant residues (Supplementary Table S3, Supplementary Figure S9) in their respective 3D structures. For the ‘Mutation list’ option, the server output is summarized as a downloadable table, and users have the option to analyze each mutation separately, similar to the analysis of a single mutation (Supplementary Figure S10).

DynaMut also generates and makes available for download pymol sessions for flexibility analysis and for inter-residue interactions for both wild-type and mutant structures to facilitate easy visualisation and figure preparation.

## VALIDATION

The performance of DynaMut was compared to well-established methods that also provide measurements of effects of single-point mutations on protein stability. All mutations from the data set described previously were submitted to each tool and the Pearson's Correlation Coefficient and Root Mean Squared Error were used to assess the comparison among all methods. Moreover, outliers were considered based on the absolute difference between predicted and actual values of ΔΔ*G*.

Since this definition can vary across the methods and for comparison purposes we defined ΔΔ*G* ≥ 0 as stabilizing and ΔΔ*G* < 0 as destabilizing. In the case that a method does not follow such definition, its results were adapted.

### Performance on cross validation

**Figure 2.**
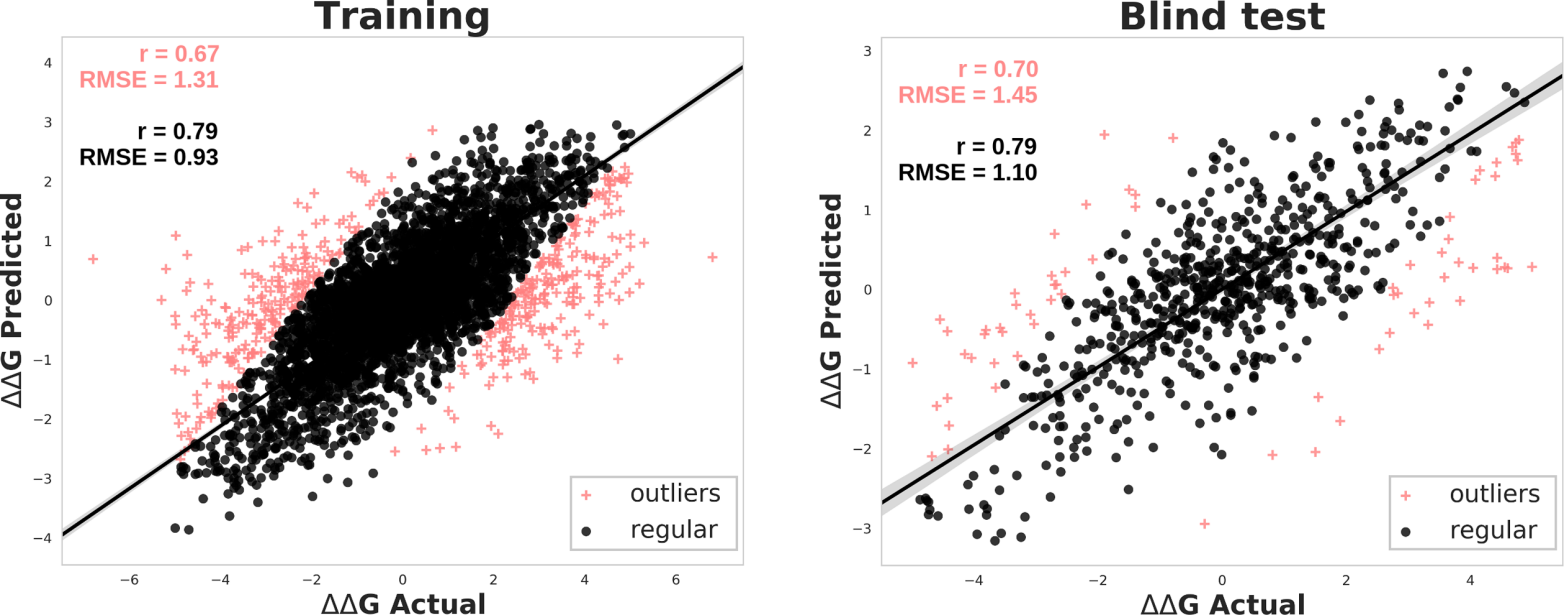
Regression analysis of the performance of DynaMut over training and blind test. Left panel shows the correlation during training and Right panel depicts the correlation between the actual values of ΔΔ*G* and the predictions of DynaMut. Pearson's correlation coefficient (*r*) and RMSE are shown. Crosses in pink show the 10% outliers. The performance results are shown on the top left of each panel. The results colored in pink are related to the entire dataset and the results colored in black were obtained after removing 10% of the outliers.

Across the full training set (forward and reverse mutations), DynaMut achieved a Pearson's correlation of *r* = 0.67, and RMSE = 1.31 kcal/mol (*r* = 0.79 and σ = 0.01 on 90% of the data) under 10-fold cross validation. This correlation was significantly higher than the individual methods used in the consensus prediction (*P*-value < 0.0001). Supplementary Table S1 on Supplementary Materials summarizes the performance for all the methods during training of DynaMut. Figure 2A shows the regression analysis for performance of DynaMut over the training set.

### Blind test

The non-redundant blind test was used to evaluate the generalization of the consensus predictions. Across the complete blind test set of 702 mutations containing both forward and hypothetical reverse mutations, DynaMut obtained a Pearson's correlation coefficient of 0.70 (RMSE = 1.45; Figure 2B). After removing 10% outliers, DynaMut achieves a correlation of up to *r* = 0.79 (RMSE = 1.10; Figure 2B). This was significantly higher (*P*-value < 0.001) than comparable methods (Table 1).

**Table 1.** Performance of DynaMut on Blind test for the 351 mutations with experimental 3D structure (forward), the 351 hypothetical reverse mutations (reverse) and the overall results for all the 702 mutations (Overall). The performance of well-established methods are also shown for comparison purposes

Method	Forward		Reverse		Overall	
	Pearson (*r*)	RMSE	Pearson (*r*)	RMSE	Pearson (*r*)	RMSE
**DynaMut**	**0.69**	**1.39**	**0.58**	**1.51**	**0.70**	**1.45**
I-Mutant 2 (46)	0.73	1.01	0.21^a^	2.55	0.49^a^	1.97
Maestro (47)	0.20^a^	2.13	0.60	2.12	0.49^a^	2.13
DUET (17)	0.75	1.05	0.27^a^	2.39	0.56^a^	1.85
SDM2 (16)	0.52^a^	1.80	0.42^a^	2.16	0.50^a^	1.99
mCSM (18)	0.76	1.09	0.23^a^	2.50	0.54^a^	1.93
ENCoM (9)	0.44^a^	1.79	−0.50^a^	2.31	0.35^a^	1.79
FoldX (48)	0.35^a^	2.33	−0.29^a^	2.23	−0.55^a^	2.32

^a^ *P*-value < 0.001 compared to DynaMut using z-test.

Looking specifically at those data points with experimental data, the original core 351 non-redundant mutations, DynaMut achieved a Pearson's correlation of *r* = 0.69 (RMSE = 1.39), significantly higher than the performance of either ENCoM, FoldX, SDM or Maestro, but lower than I-Mutant2, DUET and mCSM (*P*-value < 0.001; Table 1). Considering the hypothetical reverse mutations alone, DynaMut significantly outperformed all other algorithms tested, achieving a Pearson's correlation of 0.58 (RMSE = 1.51; Table 1).

Previous studies have highlighted that many machine learning based structural approaches are unbalanced, and can less accurately identify stabilizing mutations (16). We therefore considered method performance across stabilizing and destabilizing mutations separately (Supplementary Table S2). Considering the destabilizing mutations alone, DynaMut has a comparable correlation coefficient but higher RMSE (1.42) than mCSM (1.02), DUET (1.04) and iMutant2 (1.07), and outperformed the other methods tested. Across the stabilizing mutations, however, DynaMut achieved a correlation of *r* = 0.51 (RMSE = 1.48), significantly higher than all comparative methods (*P* < 0.01; Supplementary Table S3). This highlights that DynaMut provides the most accurate and balanced approach for the prediction of both destabilizing and stabilizing mutations.

## CONCLUSION

Here, we present DynaMut, an integrated computational method that provides users with easy access to powerful and insightful analysis of protein motions and their changes upon mutation. By consolidating these insights with our graph-based signatures, DynaMut is able to accurately assess the effects of missense mutations on protein stability. This consensus approach allows for the more accurate and reliable prediction of both stabilizing and destabilizing mutations. DynaMut is a valuable tool for a wide variety of applications, ranging from protein functional analysis, optimization of stability and understanding the role of mutations in diseases. The method is freely available as a user friendly and easy to use web server at http://biosig.unimelb.edu.au/dynamut/.

## Supplementary Material

Supplementary Data

## References

[1] Karplus M., Kuriyan J. Molecular dynamics and protein function. Proc. Natl. Acad. Sci. U.S.A. 2005; 102:6679–6685. 15870208 10.1073/pnas.0408930102 PMC1100762

[2] Jubb H., Blundell T.L., Ascher D.B. Flexibility and small pockets at protein-protein interfaces: New insights into druggability. Prog. Biophys. Mol. Biol. 2015; 119:2–9. 25662442 10.1016/j.pbiomolbio.2015.01.009 PMC4726663

[3] Albanaz A.T.S., Rodrigues C.H.M., Pires D.E.V., Ascher D.B. Combating mutations in genetic disease and drug resistance: understanding molecular mechanisms to guide drug design. Expert Opin. Drug Discov. 2017; 12:553–563. 28490289 10.1080/17460441.2017.1322579

[4] Ascher D.B., Wielens J., Nero T.L., Doughty L., Morton C.J., Parker M.W. Potent hepatitis C inhibitors bind directly to NS5A and reduce its affinity for RNA. Sci. Rep. 2014; 4:4765. 24755925 10.1038/srep04765 PMC3996483

[5] Ramdzan Y.M., Trubetskov M.M., Ormsby A.R., Newcombe E.A., Sui X., Tobin M.J., Bongiovanni M.N., Gras S.L., Dewson G., Miller J.M.L. Huntingtin inclusions trigger cellular quiescence, deactivate apoptosis, and lead to delayed necrosis. Cell Rep. 2017; 19:919–927. 28467905 10.1016/j.celrep.2017.04.029

[6] Soardi F.C., Machado-Silva A., Linhares N.D., Zheng G., Qu Q., Pena H.B., Martins T.M.M., Vieira H.G.S., Pereira N.B., Melo-Minardi R.C. Familial STAG2 germline mutation defines a new human cohesinopathy. NPJ Genom. Med. 2017; 2:7. 29263825 10.1038/s41525-017-0009-4 PMC5677968

[7] Trezza A., Bernini A., Langella A., Ascher D.B., Pires D.E.V., Sodi A., Passerini I., Pelo E., Rizzo S., Niccolai N. A computational approach from gene to structure analysis of the human ABCA4 transporter involved in genetic retinal diseases. Invest. Ophthalmol. Vis. Sci. 2017; 58:5320–5328. 29049734 10.1167/iovs.17-22158

[8] Grant B.J., Rodrigues A.P., ElSawy K.M., McCammon J.A., Caves L.S. Bio3d: an R package for the comparative analysis of protein structures. Bioinformatics. 2006; 22:2695–2696. 16940322 10.1093/bioinformatics/btl461

[9] Frappier V., Najmanovich R.J. A coarse-grained elastic network atom contact model and its use in the simulation of protein dynamics and the prediction of the effect of mutations. PLoS Comput. Biol. 2014; 10:e1003569. 24762569 10.1371/journal.pcbi.1003569 PMC3998880

[10] Lopez-Blanco J.R., Aliaga J.I., Quintana-Orti E.S., Chacon P. iMODS: internal coordinates normal mode analysis server. Nucleic Acids Res. 2014; 42:W271–W276. 24771341 10.1093/nar/gku339 PMC4086069

[11] Camps J., Carrillo O., Emperador A., Orellana L., Hospital A., Rueda M., Cicin-Sain D., D’Abramo M., Gelpi J.L., Orozco M. FlexServ: an integrated tool for the analysis of protein flexibility. Bioinformatics. 2009; 25:1709–1710. 19429600 10.1093/bioinformatics/btp304

[12] Suhre K., Sanejouand Y.H. ElNemo: a normal mode web server for protein movement analysis and the generation of templates for molecular replacement. Nucleic Acids Res. 2004; 32:W610–W614. 15215461 10.1093/nar/gkh368 PMC441506

[13] Eyal E., Lum G., Bahar I. The anisotropic network model web server at 2015 (ANM 2.0). Bioinformatics. 2015; 31:1487–1489. 25568280 10.1093/bioinformatics/btu847 PMC4410662

[14] Tiwari S.P., Fuglebakk E., Hollup S.M., Skjaerven L., Cragnolini T., Grindhaug S.H., Tekle K.M., Reuter N. WEBnm@ v2.0: Web server and services for comparing protein flexibility. BMC Bioinformatics. 2014; 15:427. 25547242 10.1186/s12859-014-0427-6 PMC4339738

[15] Dehouck Y., Grosfils A., Folch B., Gilis D., Bogaerts P., Rooman M. Fast and accurate predictions of protein stability changes upon mutations using statistical potentials and neural networks: PoPMuSiC-2.0. Bioinformatics. 2009; 25:2537–2543. 19654118 10.1093/bioinformatics/btp445

[16] Pandurangan A.P., Ochoa-Montano B., Ascher D.B., Blundell T.L. SDM: a server for predicting effects of mutations on protein stability. Nucleic Acids Res. 2017; 45:W229–W235. 28525590 10.1093/nar/gkx439 PMC5793720

[17] Pires D.E., Ascher D.B., Blundell T.L. DUET: a server for predicting effects of mutations on protein stability using an integrated computational approach. Nucleic Acids Res. 2014; 42:W314–W319. 24829462 10.1093/nar/gku411 PMC4086143

[18] Pires D.E., Ascher D.B., Blundell T.L. mCSM: predicting the effects of mutations in proteins using graph-based signatures. Bioinformatics. 2014; 30:335–342. 24281696 10.1093/bioinformatics/btt691 PMC3904523

[19] Kumar M.D., Bava K.A., Gromiha M.M., Prabakaran P., Kitajima K., Uedaira H., Sarai A. ProTherm and ProNIT: thermodynamic databases for proteins and protein-nucleic acid interactions. Nucleic Acids Res. 2006; 34:D204–D206. 16381846 10.1093/nar/gkj103 PMC1347465

[20] Potapov V., Cohen M., Schreiber G. Assessing computational methods for predicting protein stability upon mutation: good on average but not in the details. Protein Eng. Des. Sel. 2009; 22:553–560. 19561092 10.1093/protein/gzp030

[21] Khan S., Vihinen M. Performance of protein stability predictors. Hum Mutat. 2010; 31:675–684. 20232415 10.1002/humu.21242

[22] Thiltgen G., Goldstein R.A. Assessing predictors of changes in protein stability upon mutation using self-consistency. PLoS One. 2012; 7:e46084. 23144695 10.1371/journal.pone.0046084 PMC3483175

[23] Pires D.E., Ascher D.B. mCSM-AB: a web server for predicting antibody-antigen affinity changes upon mutation with graph-based signatures. Nucleic Acids Res. 2016; 44:W469–W473. 27216816 10.1093/nar/gkw458 PMC4987957

[24] Pires D.E., Ascher D.B. mCSM-NA: predicting the effects of mutations on protein-nucleic acids interactions. Nucleic Acids Res. 2017; 45:W241–W246. 28383703 10.1093/nar/gkx236 PMC5570212

[25] Tasumi M., Takeuchi H., Ataka S., Dwivedi A.M., Krimm S. Normal vibrations of proteins: glucagon. Biopolymers. 1982; 21:711–714. 7066480 10.1002/bip.360210318

[26] Go N., Noguti T., Nishikawa T. Dynamics of a small globular protein in terms of low-frequency vibrational modes. Proc. Natl. Acad. Sci. U.S.A. 1983; 80:3696–3700. 6574507 10.1073/pnas.80.12.3696 PMC394117

[27] Levitt M., Sander C., Stern P.S. Protein normal-mode dynamics: trypsin inhibitor, crambin, ribonuclease and lysozyme. J. Mol. Biol. 1985; 181:423–447. 2580101 10.1016/0022-2836(85)90230-x

[28] Bahar I., Lezon T.R., Bakan A., Shrivastava I.H. Normal mode analysis of biomolecular structures: functional mechanisms of membrane proteins. Chem. Rev. 2010; 110:1463–1497. 19785456 10.1021/cr900095e PMC2836427

[29] Hinsen K. Analysis of domain motions by approximate normal mode calculations. Proteins. 1998; 33:417–429. 9829700 10.1002/(sici)1097-0134(19981115)33:3<417::aid-prot10>3.0.co;2-8

[30] Jafri M., Wake N.C., Ascher D.B., Pires D.E., Gentle D., Morris M.R., Rattenberry E., Simpson M.A., Trembath R.C., Weber A. Germline mutations in the CDKN2B tumor suppressor gene predispose to renal cell carcinoma. Cancer Discov. 2015; 5:723–729. 25873077 10.1158/2159-8290.CD-14-1096

[31] Usher J.L., Ascher D.B., Pires D.E., Milan A.M., Blundell T.L., Ranganath L.R. Analysis of HGD gene mutations in patients with alkaptonuria from the United Kingdom: Identification of novel mutations. JIMD Rep. 2015; 24:3–11. 25681086 10.1007/8904_2014_380 PMC4582018

[32] Kano F.S., Souza-Silva F.A., Torres L.M., Lima B.A., Sousa T.N., Alves J.R., Rocha R.S., Fontes C.J., Sanchez B.A., Adams J.H. The presence, persistence and functional properties of plasmodium vivax duffy binding protein II antibodies are influenced by HLA class II allelic variants. PLoS Negl. Trop. Dis. 2016; 10:e0005177. 27959918 10.1371/journal.pntd.0005177 PMC5154503

[33] Nemethova M., Radvanszky J., Kadasi L., Ascher D.B., Pires D.E., Blundell T.L., Porfirio B., Mannoni A., Santucci A., Milucci L. Twelve novel HGD gene variants identified in 99 alkaptonuria patients: focus on ‘black bone disease’ in Italy. E.ur J. Hum. Genet. 2016; 24:66–72. 10.1038/ejhg.2015.60 PMC4795215 25804398

[34] Phelan J., Coll F., McNerney R., Ascher D.B., Pires D.E., Furnham N., Coeck N., Hill-Cawthorne G.A., Nair M.B., Mallard K. Mycobacterium tuberculosis whole genome sequencing and protein structure modelling provides insights into anti-tuberculosis drug resistance. BMC Med. 2016; 14:31. 27005572 10.1186/s12916-016-0575-9 PMC4804620

[35] White R.R., Ponsford A.H., Weekes M.P., Rodrigues R.B., Ascher D.B., Mol M., Selkirk M.E., Gygi S.P., Sanderson C.M., Artavanis-Tsakonas K. Ubiquitin-Dependent modification of skeletal muscle by the parasitic nematode, trichinella spiralis. PLoS Pathog. 2016; 12:e1005977. 27870901 10.1371/journal.ppat.1005977 PMC5117777

[36] Casey R.T., Ascher D.B., Rattenberry E., Izatt L., Andrews K.A., Simpson H.L., Challis B., Park S.M., Bulusu V.R., Lalloo F. SDHA related tumorigenesis: a new case series and literature review for variant interpretation and pathogenicity. Mol. Genet. Genomic Med. 2017; 5:237–250. 28546994 10.1002/mgg3.279 PMC5441402

[37] Pandurangan A.P., Ascher D.B., Thomas S.E., Blundell T.L. Genomes, structural biology and drug discovery: combating the impacts of mutations in genetic disease and antibiotic resistance. Biochem. Soc. Trans. 2017; 45:303–311. 28408471 10.1042/BST20160422 PMC5390495

[38] Park Y., Pacitto A., Bayliss T., Cleghorn L.A., Wang Z., Hartman T., Arora K., Ioerger T.R., Sacchettini J., Rizzi M. Essential but not Vulnerable: Indazole sulfonamides targeting inosine monophosphate dehydrogenase as potential leads against mycobacterium tuberculosis. ACS Infect. Dis. 2017; 3:18–33. 27704782 10.1021/acsinfecdis.6b00103 PMC5972394

[39] Singh V., Donini S., Pacitto A., Sala C., Hartkoorn R.C., Dhar N., Keri G., Ascher D.B., Mondesert G., Vocat A. The inosine monophosphate dehydrogenase, GuaB2, is a vulnerable new bactericidal drug target for tuberculosis. ACS Infect. Dis. 2017; 3:5–17. 27726334 10.1021/acsinfecdis.6b00102 PMC5241705

[40] Pires D.E., Ascher D.B. CSM-lig: a web server for assessing and comparing protein-small molecule affinities. Nucleic Acids Res. 2016; 44:W557–W561. 27151202 10.1093/nar/gkw390 PMC4987933

[41] Pires D.E., Blundell T.L., Ascher D.B. Platinum: a database of experimentally measured effects of mutations on structurally defined protein-ligand complexes. Nucleic Acids Res. 2015; 43:D387–D391. 25324307 10.1093/nar/gku966 PMC4384026

[42] Pires D.E., Blundell T.L., Ascher D.B. mCSM-lig: quantifying the effects of mutations on protein-small molecule affinity in genetic disease and emergence of drug resistance. Sci. Rep. 2016; 6:29575. 27384129 10.1038/srep29575 PMC4935856

[43] Pires D.E., Chen J., Blundell T.L., Ascher D.B. In silico functional dissection of saturation mutagenesis: Interpreting the relationship between phenotypes and changes in protein stability, interactions and activity. Sci. Rep. 2016; 6:19848. 26797105 10.1038/srep19848 PMC4726175

[44] Breiman L. Random Forests. Mach. Learn. 2001; 45:5–32.

[45] Jubb H.C., Higueruelo A.P., Ochoa-Montano B., Pitt W.R., Ascher D.B., Blundell T.L. Arpeggio: A web server for calculating and visualising interatomic interactions in protein structures. J. Mol. Biol. 2017; 429:365–371. 27964945 10.1016/j.jmb.2016.12.004 PMC5282402

[46] Capriotti E., Fariselli P., Casadio R. I-Mutant2.0: predicting stability changes upon mutation from the protein sequence or structure. Nucleic Acids Res. 2005; 33:W306–W310. 15980478 10.1093/nar/gki375 PMC1160136

[47] Laimer J., Hofer H., Fritz M., Wegenkittl S., Lackner P. MAESTRO–multi agent stability prediction upon point mutations. BMC Bioinformatics. 2015; 16:116. 25885774 10.1186/s12859-015-0548-6 PMC4403899

[48] Schymkowitz J., Borg J., Stricher F., Nys R., Rousseau F., Serrano L. The FoldX web server: an online force field. Nucleic Acids Res. 2005; 33:W382–W388. 15980494 10.1093/nar/gki387 PMC1160148

