# Supplementary material for: DynaMut: predicting the impact of mutations on protein conformation, flexibility and stability
Source: Nucleic Acids Res. 2018 Apr 30;46(Web Server issue):W350–5. doi: 10.1093/nar/gky300 (PMC6031064; doi:10.1093/nar/gky300)
Supplement: Supplementary Data [file gky300_supplemental_files.docx]

# SUPPLEMENTARY MATERIAL

**DynaMut: analysis and prediction of protein stability changes upon mutation using Normal Mode Analysis**

Carlos H.M. Rodrigues^1^, Douglas E.V. Pires^3,^*, David B. Ascher^1,2,3,^*

^1^Department of Biochemistry and Molecular Biology, Bio21 Institute, University of Melbourne;

^2^Department of Biochemistry, University of Cambridge;

^3^Instituto René Rachou, Fundação Oswaldo Cruz

*To whom correspondence should be addressed D.B.A. Tel: +61 90354794; Email: [david.ascher@unimelb.edu.au](mailto:david.ascher@unimelb.edu.au) or [da382@cam.ac.uk](mailto:da382@cam.ac.uk). Correspondence may also be addressed to D.E.V.P. [douglas.pires@minas.fiocruz.br](mailto:douglas.pires@minas.fiocruz.br).

# CONSENSUS PREDICTION COMPONENTS

**Bio3D**

Bio3D is a R package that contains utilities that helps one to process, organize and explore protein structure and sequence data. Among other features available, Bio3D provides the ability to read, write and process biomolecular structure, sequence and dynamics trajectory data; perform ensemble normal mode analysis on large structure sets to explore evolutionary dynamics and structure sets to explore evolutionary dynamics and structure dependent protein flexibility; and also various utility functions are provided to enable the statistical and graphical power of the R environment when working with biological sequence and structural data (1). The package source code is freely available at <https://bitbucket.org/Grantlab/bio3d/>.

**ENCoM**

ENCoM is an Elastic Network Contact Model that employs a potential energy function and includes a pairwise atom-type non-bonded interaction term to add an extra layer of information regarding the effect of the specific nature of amino acids on dynamics within the context of NMA (2). ENCoM tries to approximate ΔΔG through the calculations of the vibrational entropy (ΔS) (3) of wild-type and mutant structures. The ΔS between two conformations (A, B) in terms of their respective sets of eigenvalues is given by:

$$\Delta S_{Vib, A\to B}= \ln\left( \frac{\prod_{n=7}^{3N} \lambda_{n,A}}{\prod_{n=7}^{3N} \lambda_{n,B}} \right)$$

where:

- $\boldsymbol{\lambda}_{\boldsymbol{n,i}}$ represents the *n*th normal mode (the first 6 modes correspond to rotational and translational degrees of freedom and because of that they are not considered on the calculations.

The source code for ENCoM is publicly available at https://github.com/NRGlab/ENCoM.

**DUET**

DUET is an integrated approach for predicting the effects of mutations on protein stability that takes advantage of two distinct techniques, SDM (4) and mCSM (5), by combining them in a consensus prediction (6). DUET unifies the results of the separate methods in an optimised predictor using Support Vector Machines (SVM) trained with Sequential Minimal Optimization (7). DUET predictions were more accurate than either method on their own. DUET is freely available as a web server at <http://biosig.unimelb.edu.au/duet>.

# EVALUATION METRICS

A set of well-established and widely used performance metrics for evaluation regression models were used to evaluate DynaMut on both 10-fold cross validation and on blind tests. These metrics include Pearson’s Coefficient of Correlation (*r)* and Root Mean Squared Error (RMSE).

**Pearson’s Coefficient of Correlation**

The Pearson correlation coefficient, also known as the product moment correlation coefficient, is a measure of the linear correlation between two variables $X$ and $Y$. The coefficient is measure on a scale with no units and can take values from -1, total negative linear correlation, to +1, total positive correlation. Values closer from 0 indicate that there is no linear correlation between $X$ and $Y$. The mathematical definition of the Pearson's Correlation Coefficient is given by the covariance of the two variables divided by the product of their standard deviations as described by the formula below (8).

$\rho_{X,Y}=\frac{cov(X,Y)}{\sigma_{X}\sigma_{Y}}$ where:

- $\boldsymbol{cov}$**(**$\boldsymbol{X}$**,**$\boldsymbol{Y}$**)** is the covariance of $\boldsymbol{X}$ and $\boldsymbol{Y}$;
- $\boldsymbol{\sigma}_{\boldsymbol{X}}$ is the standard deviation of the variable $\boldsymbol{X}$;
- $\boldsymbol{\sigma}_{\boldsymbol{Y}}$ is the standard deviation of the variable $\boldsymbol{Y}$;

**Root Mean Squared Error**

Root Mean Squared Error (RMSE) is the standard deviation of the predictions errors. This measure indicates how concentrated the predicted data points are from the line of best fit which represents the ideal perfect correlation between the actual observed values ($Y$) and the predicted values ($\hat{Y}$) (9). RMSE is described by the formula below:

$$RMSE= \sqrt{\frac{1}{n}\sum_{i=1}^{n} {(Y_{i}-\hat{Y}_{i})}^{2}}$$

where:

- $\boldsymbol{n}$ is the total number of instances;
- ${\boldsymbol{(}\boldsymbol{Y}_{\boldsymbol{i}}\boldsymbol{-}{\hat{\boldsymbol{Y}}}_{\boldsymbol{i}}\boldsymbol{)}}^{\boldsymbol{2}}$ represents the squared errors between actual observed values and the predictions;

**MACHINE LEARNING**

The Machine learning task used on this work was implemented on the Weka Tool Kit (10).

**Random Forest**

The Random Forest algorithm uses a set of decision tree predictors in a way that each tree relies on the values of a random vector sampled independently and with the same distribution for all trees in the set. The generalization error for forests converge to a limit as the number of trees in the set becomes large (11).

This is a fast and easy to implement algorithm that produces highly accurate predictions and can handle a large number of input variables with low overfitting rates. Since all the trees are built from scratch without any previous information on the other trees (reason why the algorithm is called Random Forest) in the forest and also the final prediction is the average of all the predictions for each tree.

**TABLES**

**Table S1** – Force Fields options for Normal Mode Analysis in DynaMut.

| Name | Description |
| --- | --- |
| C-alpha (12) | Force field derived from fitting to the Amber94 all-atom potential. |
| ANM (13) | Anisotropic Network Model uses a simplified spring force constant based on the pair-wise distance. |
| pfANM (14) | parameter-free Anisotropic Network Model is variant from the ANM force field with interactions that fall off with the square of the distance. |
| REACH (15) | Realistic Extension Algorithm via Covariance Hessian is parameterized based on variance-covariance matrices obtained from MD simulations. |
| sdENM (16) | This force field employs residue specific spring force constants and it has been parameterized through a statistical analysis of 1500 NMR ensembles. |

**Table S2** – Performance evaluation of DynaMut on training and comparison with other methods.

| Methods | Pearson (r) | RMSE |
| --- | --- | --- |
| **DynaMut** | **0.67** | **1.31** |
| DUET (6) | 0.41* | 1.79 |
| SDM2 (4) | 0.42* | 1.93 |
| mCSM (5) | 0.40* | 1.83 |
| ENCoM (2) | 0.05* | 5.13 |
| FoldX (17) | -0.05* | 4.37 |

** p-value < 0.001 compared to DynaMut using z-test.*

**Table S3** – Performance evaluation of DynaMut on identifying stabilizing mutations and comparison with other methods.

| Method | Stabilising | | Destabilizing | |
| --- | --- | --- | --- | --- |
|  | Person (*r*) | RMSE | Pearson (*r*) | RMSE |
| **DynaMut** | **0.51** | **1.48** | **0.61** | **1.42** |
| I-Mutant (18) | 0.07* | 2.57 | 0.57 | 1.07 |
| Maestro (19) | 0.43 | 2.22 | 0.45* | 2.13 |
| DUET (6) | 0.13* | 2.4 | 0.64 | 1.04 |
| SDM2 (4) | 0.26* | 2.15 | 0.4* | 1.83 |
| mCSM (5) | 0.12* | 2.53 | 0.63 | 1.02 |
| ENCoM (2) | 0.37* | 1.84 | 0.03* | 4.36 |
| FoldX (17) | -0.37* | 2.34 | -0.03* | 5.21 |

** p-value < 0.01 compared to DynaMut using z-test.*

**FIGURES**


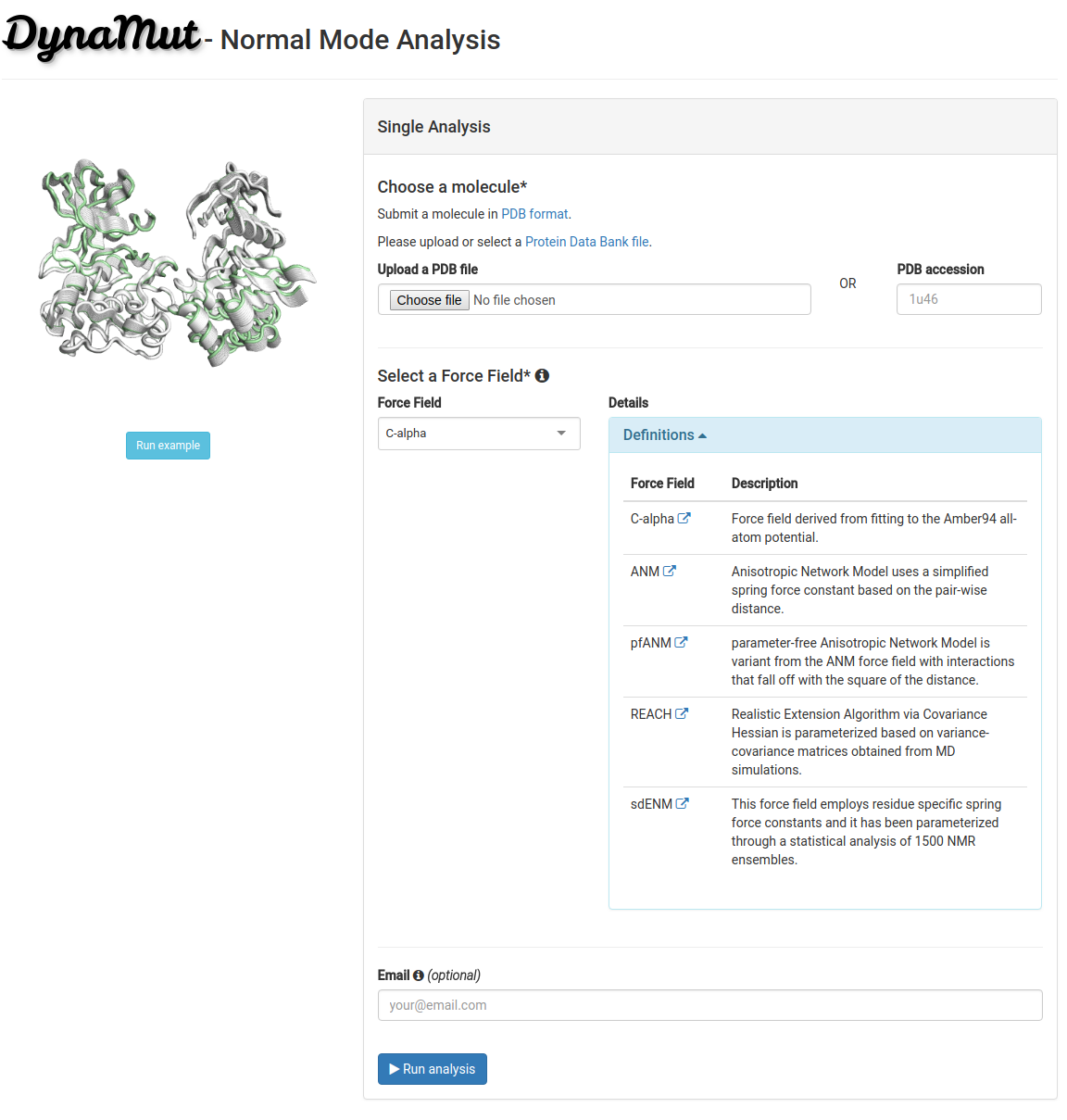


**Figure S1** - DynaMut normal mode analysis input page. For the protein dynamics analysis, the server requires the user to input a protein structure by either uploading a file in PDB format or by providing the 4-letter accession code for any entry on the PDB database. In addition, users, are required to specify a force field that will describe the interactions between the atoms of the structure for the normal mode analysis.


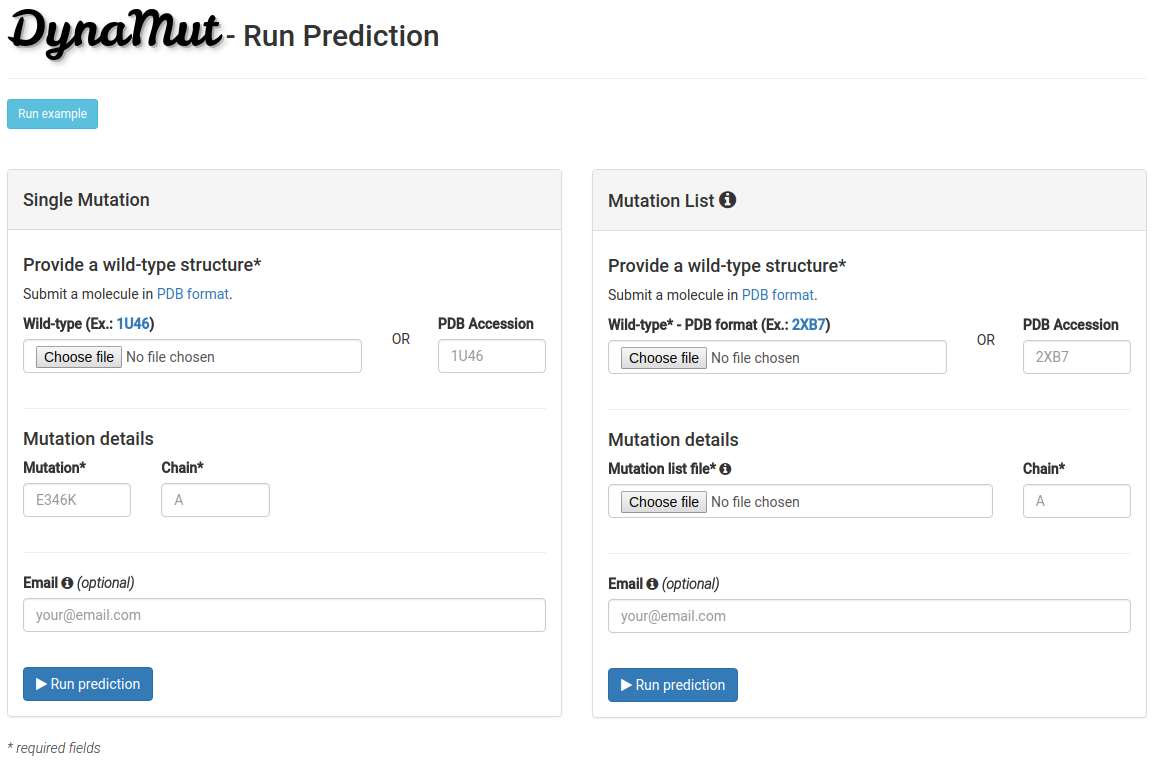


**Figure S2** - DynaMut prediction input page. For assessing effects of mutations on protein dynamics and stability two different input options are available. The "Single mutation" option requires the user to provide a PDB file or PDB accession code, the point mutation specified as a string containing the wild-type residue one-letter code, its corresponding residue number and the mutant residue one-letter code. The "Mutation list" option allows users to upload a list of mutations in a file for batch processing. For both input options the user also is asked to specify the chain identifier in which the wild-type residue is located.


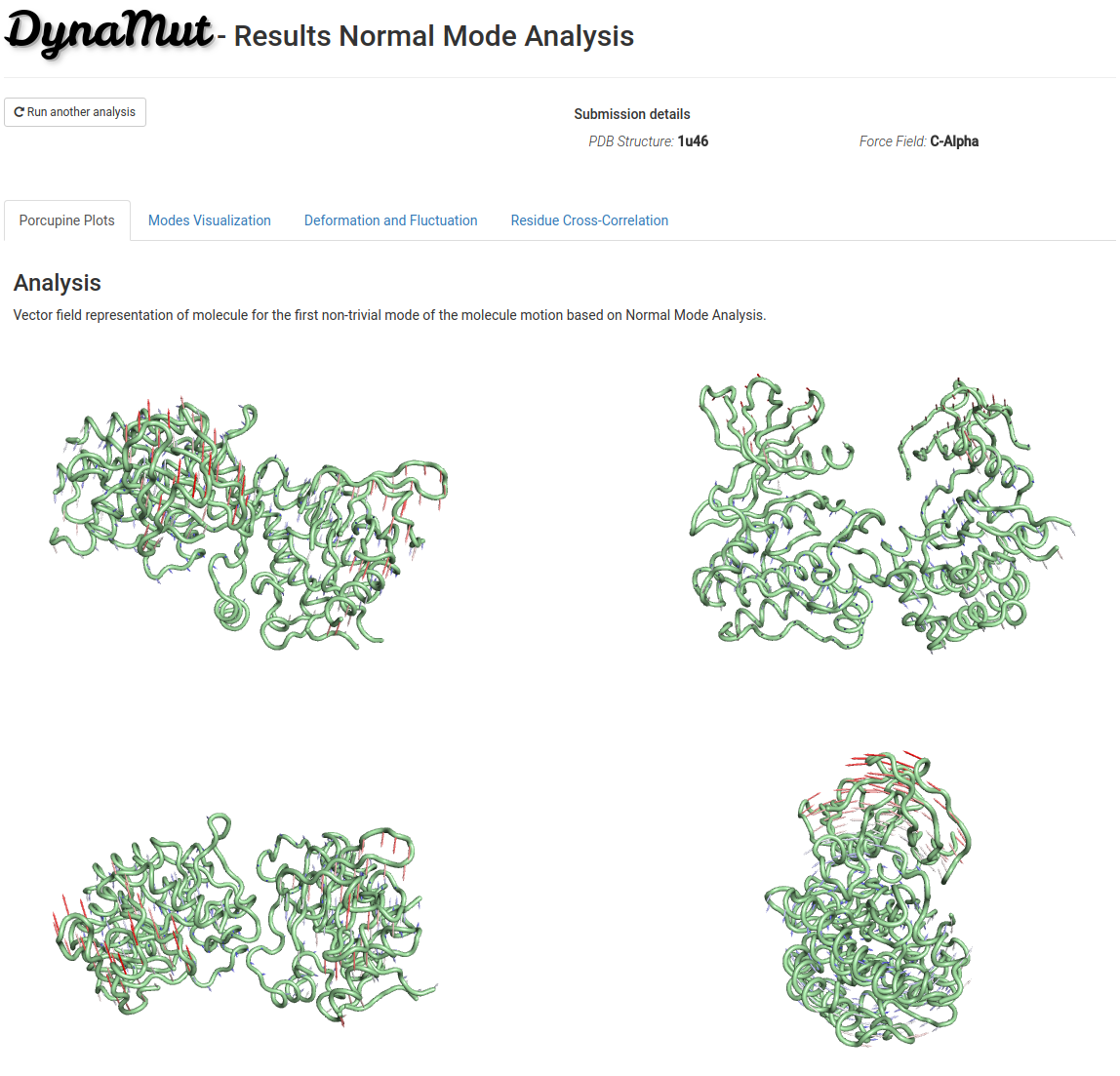


**Figure S3** - Porcupine Plots on DynaMut Normal Mode Analysis output page for the example (PDB: 1U46 – Tyrosine Kinase ACK1). Protein kinase transition state from active to inactive and vice-versa requires that the protein presents a minimum flexibility no matter if the kinase is activated or not. This transitional state is directly affected by the molecule flexibility.


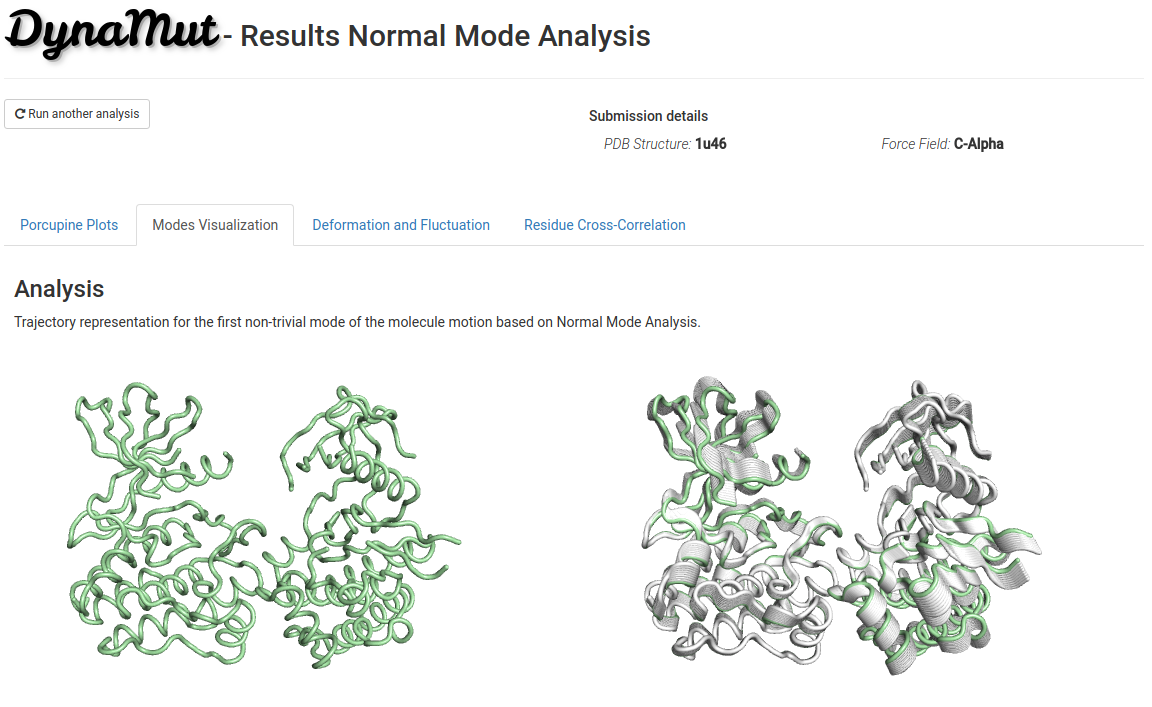


**Figure S4** - Modes Visualisation on DynaMut Normal Mode Analysis output page.


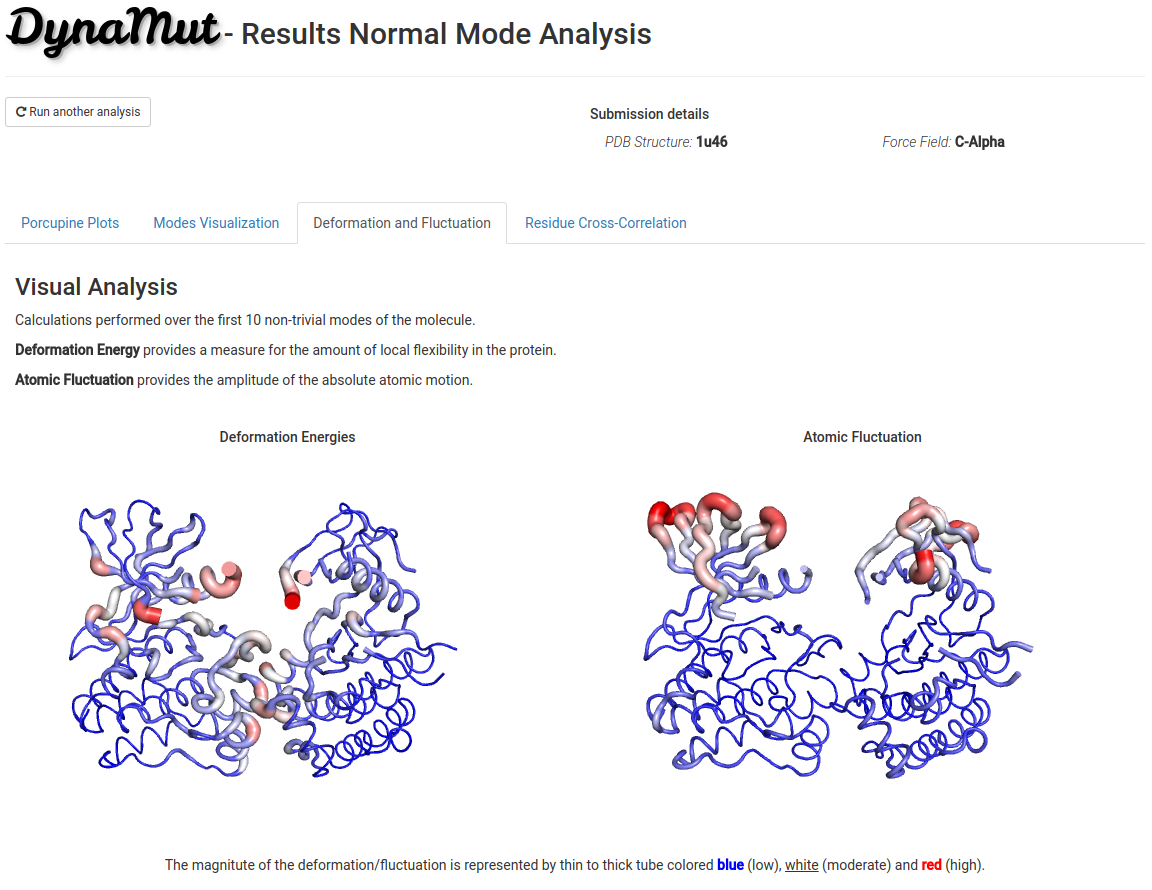


**Figure S5** - Deformation energies and atomic fluctuation on DynaMut Normal Mode Analysis output page.


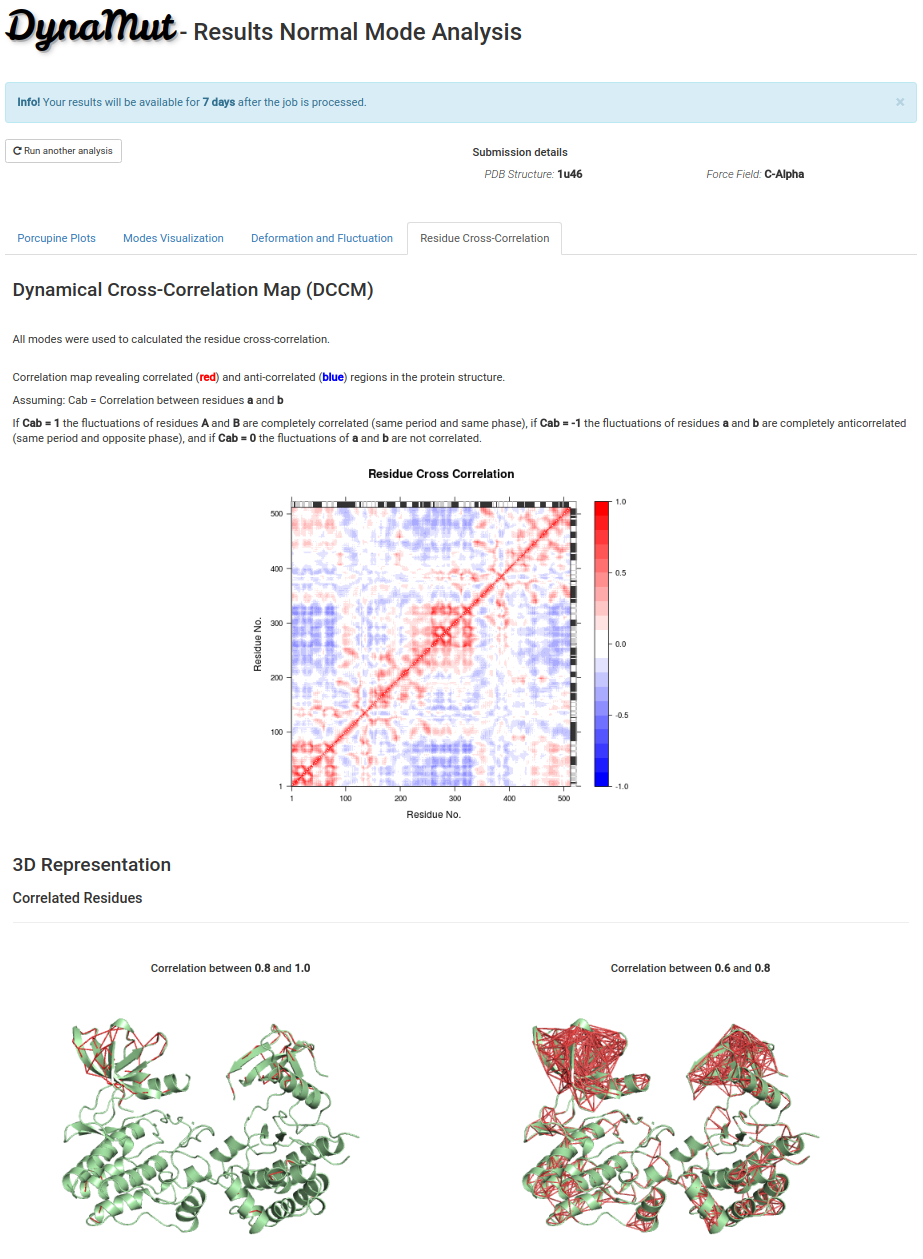


**Figure S6** - Residue cross-correlation on DynaMut Normal Mode Analysis output page.


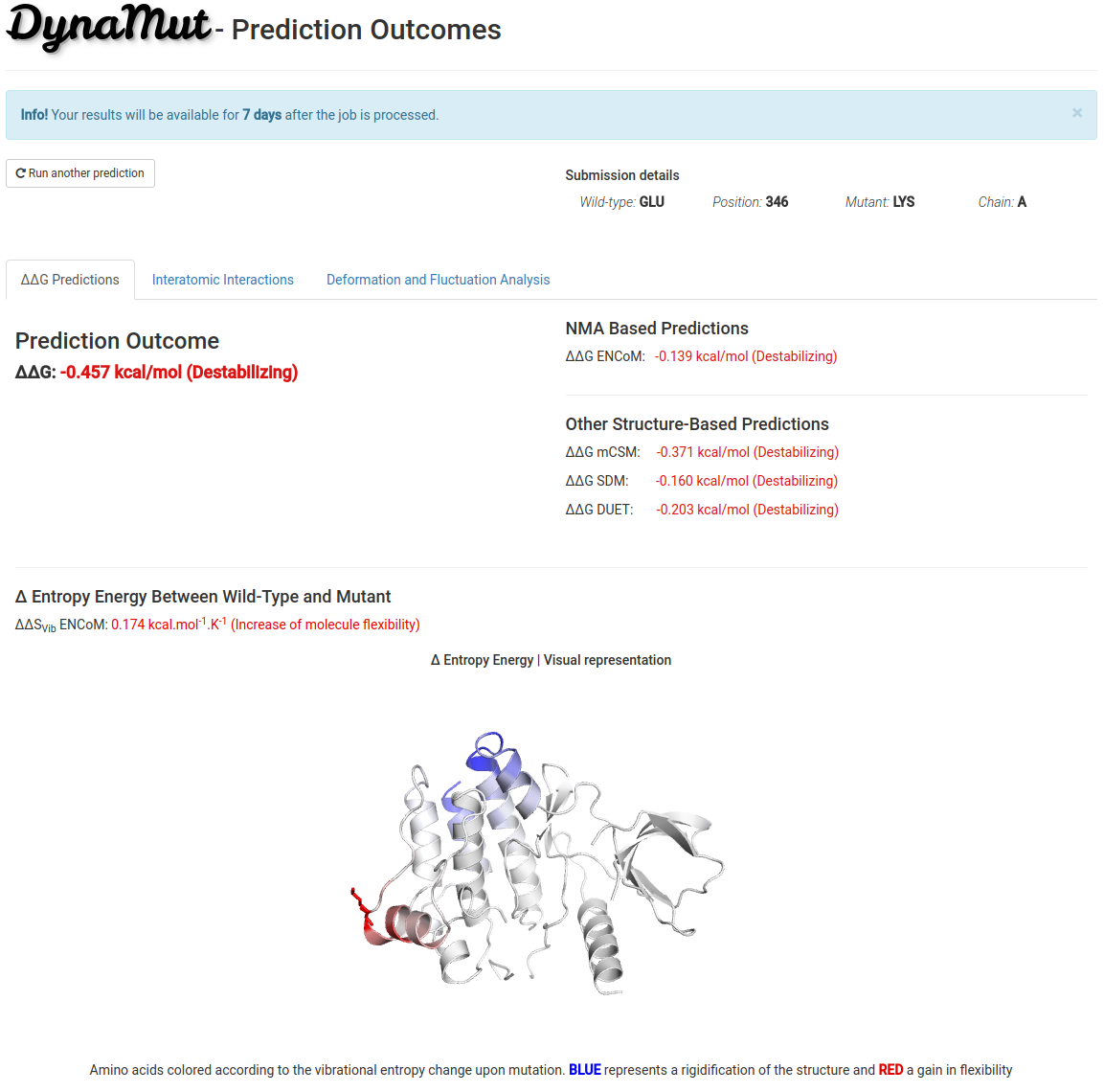


**Figure S7** - Mutation effect prediction on DynaMut Prediction output page for the example (PDB: 1U46 – Tyrosine Kinase ACK1). The mechanisms by which the activating mutations affect kinases are associated with a restriction in the transition from active to inactive, resulting in one conformational state being favoured. This transitional state is directly affected by the molecule flexibility. The ΔΔG prediction outcome is shown on the top left of the page. Results for other predictive tools (NMA based and Other Structure-based approaches) are also displayed. Visual representation of the Δ Entropy Energy in which the amino acids were coloured according to the vibrational entropy change upon mutation is shown on the bottom. Blue regions indicate rigidification and red a gain in flexibility.


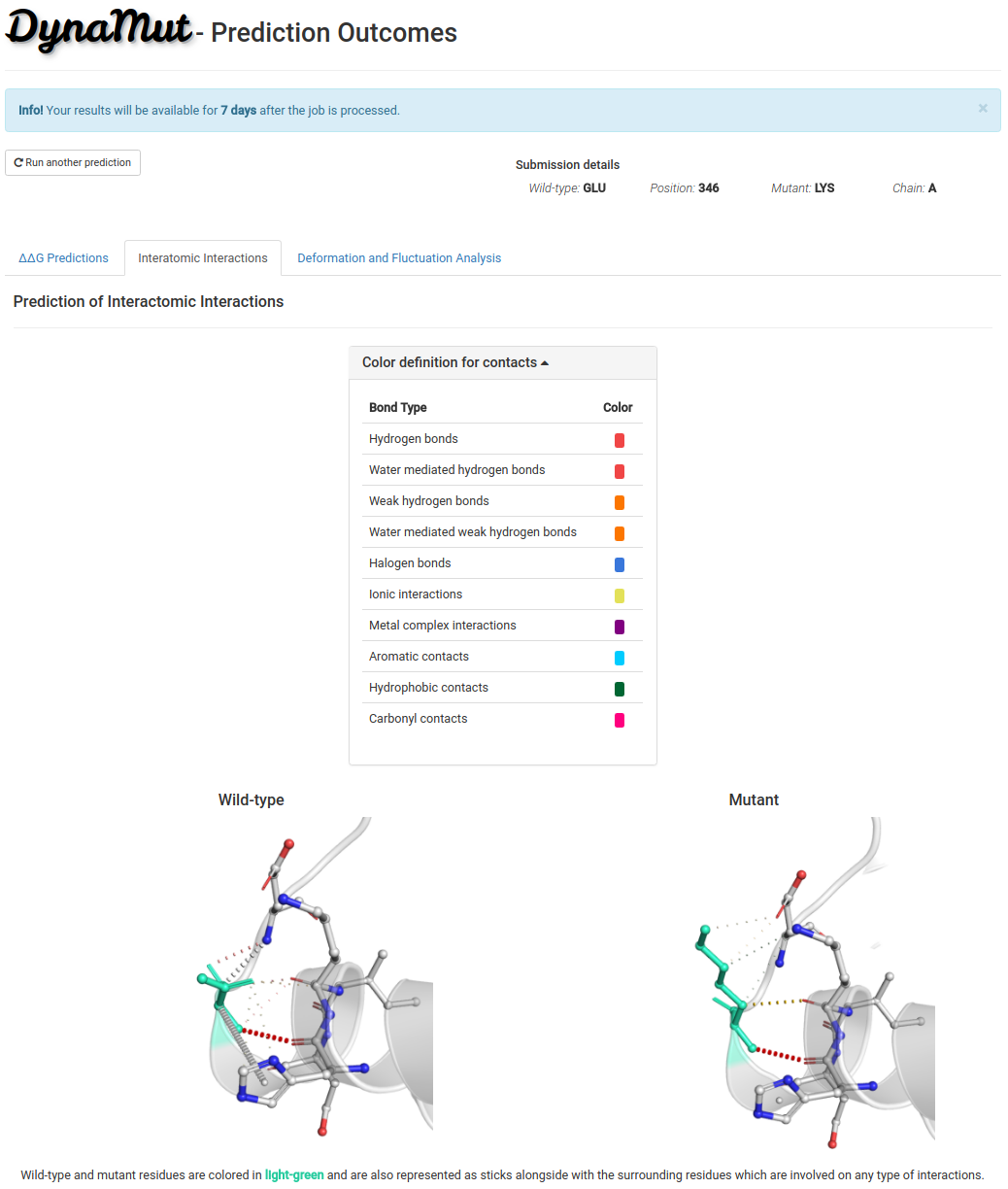


**Figure S8** - Interatomic Interactions predictions of wild-type and mutant residues on output page of DynaMut. Wild-type and mutant residues are coloured in light-green and are also represented as sticks. A table with the colour definitions for each type of interaction is shown on top.


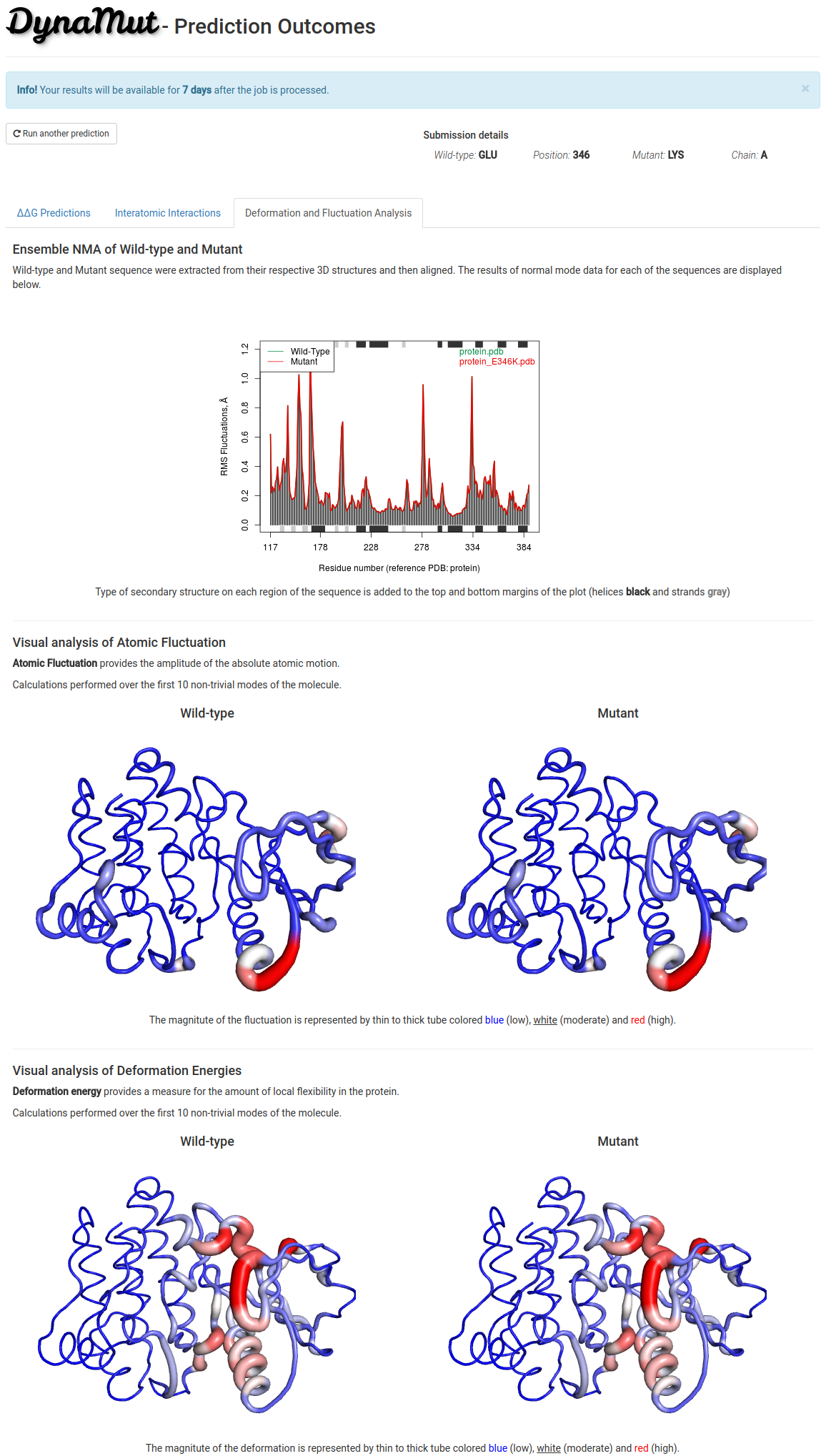


**Figure S9** - Atomic fluctuation and deformation energies of the wild-type and mutant structures on output page of DynaMut. Wild-type and Mutant sequence were extracted from their respective 3D structures and then aligned. The results of normal mode data for each of the sequences are displayed on top. Visual representation of atomic fluctuation and deformation energies for wild-type (left) and mutant (right) are shown below. The magnitude of the fluctuation and deformation is represented by thin to thick tube coloured blue (low), white (moderate) and red (high).


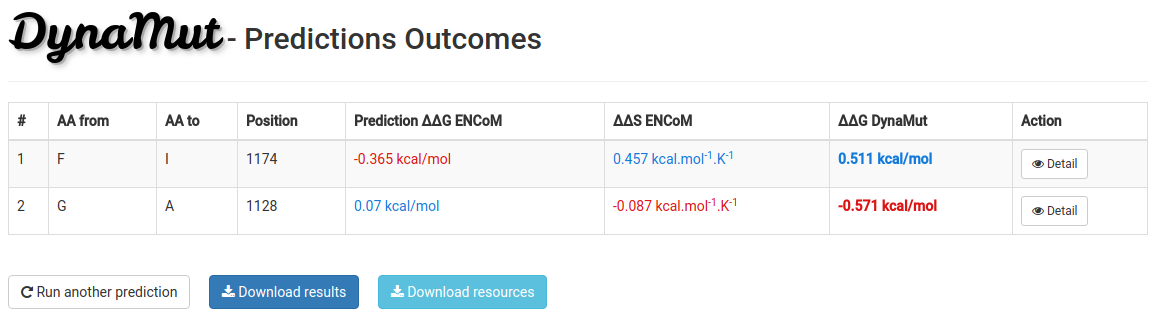


**Figure S10** – Results page of DynaMut for the Mutation list option. The server output is summarised as a downloadable table, and users have the option to analyse each mutation separately, similar to the analysis of a single mutation, by clicking on the “Detail” button of each mutation on the row. All resources generated on the analysis are also available for download.

**References**

1. Grant, B.J., Rodrigues, A.P., ElSawy, K.M., McCammon, J.A. and Caves, L.S. (2006) Bio3d: an R package for the comparative analysis of protein structures. *Bioinformatics*, **22**, 2695-2696.

<http://www.ncbi.nlm.nih.gov/pubmed/16940322>

<http://dx.doi.org/10.1093/bioinformatics/btl461>

2. Frappier, V. and Najmanovich, R.J. (2014) A coarse-grained elastic network atom contact model and its use in the simulation of protein dynamics and the prediction of the effect of mutations. *PLoS Comput Biol*, **10**, e1003569.

<http://www.ncbi.nlm.nih.gov/pubmed/24762569>

<http://dx.doi.org/10.1371/journal.pcbi.1003569>

3. Karplus, M. and Kushick, J.N. (1981) Method for estimating the configurational entropy of macromolecules. *Macromolecules*, **14**, 325-332.

<http://dx.doi.org/10.1021/ma50003a019>

4. Pandurangan, A.P., Ochoa-Montano, B., Ascher, D.B. and Blundell, T.L. (2017) SDM: a server for predicting effects of mutations on protein stability. *Nucleic Acids Res*, **45**, W229-W235.

<http://www.ncbi.nlm.nih.gov/pubmed/28525590>

<http://dx.doi.org/10.1093/nar/gkx439>

5. Pires, D.E., Ascher, D.B. and Blundell, T.L. (2014) mCSM: predicting the effects of mutations in proteins using graph-based signatures. *Bioinformatics*, **30**, 335-342.

<http://www.ncbi.nlm.nih.gov/pubmed/24281696>

<http://dx.doi.org/10.1093/bioinformatics/btt691>

6. Pires, D.E., Ascher, D.B. and Blundell, T.L. (2014) DUET: a server for predicting effects of mutations on protein stability using an integrated computational approach. *Nucleic Acids Res*, **42**, W314-319.

<http://www.ncbi.nlm.nih.gov/pubmed/24829462>

<http://dx.doi.org/10.1093/nar/gku411>

7. Shevade, S.K., Keerthi, S.S., Bhattacharyya, C. and Murthy, K.K. (2000) Improvements to the SMO algorithm for SVM regression. *IEEE Trans Neural Netw*, **11**, 1188-1193.

<http://www.ncbi.nlm.nih.gov/pubmed/18249845>

<http://dx.doi.org/10.1109/72.870050>

8. Sedgwick, P. (2012) Pearson’s correlation coefficient. *BMJ : British Medical Journal*, **345**.

<http://dx.doi.org/10.1136/bmj.e4483>

9. Hyndman, R.J. and Koehler, A.B. (2006) Another look at measures of forecast accuracy. *International Journal of Forecasting*, **22**, 679-688.

<http://dx.doi.org/https://doi.org/10.1016/j.ijforecast.2006.03.001>

10. Hall, M., Frank, E., Holmes, G., Pfahringer, B., Reutemann, P. and Witten, I.H. (2009) The WEKA data mining software: an update. *SIGKDD Explor. Newsl.*, **11**, 10-18.

<http://dx.doi.org/10.1145/1656274.1656278>

<http://www.ncbi.nlm.nih.gov/pmc/articles/1656278>

11. (2007) In Ilias, M., Kostas, K., Manolis, W. and John, S. (eds.). IOS Press.

12. Hayward, S., Kitao, A. and Go, N. (1995) Harmonicity and anharmonicity in protein dynamics: a normal mode analysis and principal component analysis. *Proteins*, **23**, 177-186.

<http://www.ncbi.nlm.nih.gov/pubmed/8592699>

<http://dx.doi.org/10.1002/prot.340230207>

13. Atilgan, A.R., Durell, S.R., Jernigan, R.L., Demirel, M.C., Keskin, O. and Bahar, I. (2001) Anisotropy of fluctuation dynamics of proteins with an elastic network model. *Biophys J*, **80**, 505-515.

<http://www.ncbi.nlm.nih.gov/pubmed/11159421>

<http://dx.doi.org/10.1016/S0006-3495(01)76033-X>

14. Yang, L., Song, G. and Jernigan, R.L. (2009) Protein elastic network models and the ranges of cooperativity. *Proc Natl Acad Sci U S A*, **106**, 12347-12352.

<http://www.ncbi.nlm.nih.gov/pubmed/19617554>

<http://dx.doi.org/10.1073/pnas.0902159106>

15. Moritsugu, K. and Smith, J.C. (2007) Coarse-grained biomolecular simulation with REACH: realistic extension algorithm via covariance Hessian. *Biophys J*, **93**, 3460-3469.

<http://www.ncbi.nlm.nih.gov/pubmed/17693469>

<http://dx.doi.org/10.1529/biophysj.107.111898>

16. Dehouck, Y. and Mikhailov, A.S. (2013) Effective harmonic potentials: insights into the internal cooperativity and sequence-specificity of protein dynamics. *PLoS Comput Biol*, **9**, e1003209.

<http://www.ncbi.nlm.nih.gov/pubmed/24009495>

<http://dx.doi.org/10.1371/journal.pcbi.1003209>

17. Schymkowitz, J., Borg, J., Stricher, F., Nys, R., Rousseau, F. and Serrano, L. (2005) The FoldX web server: an online force field. *Nucleic Acids Res*, **33**, W382-388.

<http://www.ncbi.nlm.nih.gov/pubmed/15980494>

<http://dx.doi.org/10.1093/nar/gki387>

18. Capriotti, E., Fariselli, P. and Casadio, R. (2005) I-Mutant2.0: predicting stability changes upon mutation from the protein sequence or structure. *Nucleic Acids Res*, **33**, W306-310.

<http://www.ncbi.nlm.nih.gov/pubmed/15980478>

<http://dx.doi.org/10.1093/nar/gki375>

19. Laimer, J., Hofer, H., Fritz, M., Wegenkittl, S. and Lackner, P. (2015) MAESTRO--multi agent stability prediction upon point mutations. *BMC Bioinformatics*, **16**, 116.

<http://www.ncbi.nlm.nih.gov/pubmed/25885774>

<http://dx.doi.org/10.1186/s12859-015-0548-6>
